# Ablation of Selenbp1 Alters Lipid Metabolism via the Pparα Pathway in Mouse Kidney

**DOI:** 10.3390/ijms22105334

**Published:** 2021-05-19

**Authors:** Yingxia Song, Atsushi Kurose, Renshi Li, Tomoki Takeda, Yuko Onomura, Takayuki Koga, Junpei Mutoh, Takumi Ishida, Yoshitaka Tanaka, Yuji Ishii

**Affiliations:** 1 Laboratory of Molecular Life Sciences, Graduate School of Pharmaceutical Sciences, Kyushu University, 3-1-1 Maidashi, Higashi-ku, Fukuoka 812-8582, Japan; songyingixa324@gmail.com (Y.S.); 9070ihsustaesoruk@gmail.com (A.K.); li-renshi@cpu.edu.cn (R.L.); tomoki-takeda@jbrc.johas.go.jp (T.T.); yonomura415@gmail.com (Y.O.); 2 Division of Pharmaceutical Cell Biology, Graduate School of Pharmaceutical Sciences, Kyushu University, 3-1-1 Maidashi, Higashi-ku, Fukuoka 812-8582, Japan; ytanaka@phar.kyushu-u.ac.jp; 3 Laboratory of Hygienic Chemistry, Daiichi University of Pharmacy, 22-1 Tamagawa-cho, Minami-ku, Fukuoka 815-8511, Japan; ta-koga@daiichi-cps.ac.jp; 4 Faculty of Pharmaceutical Sciences, Sanyo-Onoda City University, Daigakudori 1-1-1, Sanyo-Onoda 756-0884, Japan; jmutoh@rs.socu.ac.jp; 5 School of Pharmacy, International University of Health and Welfare Fukuoka, Ohkawa, Fukuoka 831-8501, Japan; ishida@iuhw.ac.jp

**Keywords:** selenium binding protein 1, peroxisome proliferator-activated receptor-alpha, Ppar, mouse, kidney, lipid metabolism, oxidative stress

## Abstract

Selenium-binding protein 1 (Selenbp1) is a 2,3,7,8-tetrechlorodibenzo-*p*-dioxin inducible protein whose function is yet to be comprehensively elucidated. As the highly homologous isoform, Selenbp2, is expressed at low levels in the kidney, it is worthwhile comparing wild-type C57BL mice and Selenbp1-deficient mice under dioxin-free conditions. Accordingly, we conducted a mouse metabolomics analysis under non-dioxin-treated conditions. DNA microarray analysis was performed based on observed changes in lipid metabolism-related factors. The results showed fluctuations in the expression of numerous genes. Real-time RT-PCR confirmed the decreased expression levels of the cytochrome P450 4a (Cyp4a) subfamily, known to be involved in fatty acid ω- and ω-1 hydroxylation. Furthermore, peroxisome proliferator-activated receptor-α (Pparα) and retinoid-X-receptor-α (Rxrα), which form a heterodimer with Pparα to promote gene expression, were simultaneously reduced. This indicated that reduced Cyp4a expression was mediated via decreased Pparα and Rxrα. In line with this finding, increased levels of leukotrienes and prostaglandins were detected. Conversely, decreased hydrogen peroxide levels and reduced superoxide dismutase (SOD) activity supported the suppression of the renal expression of Sod1 and Sod2 in Selenbp1-deficient mice. Therefore, we infer that ablation of Selenbp1 elicits oxidative stress caused by increased levels of superoxide anions, which alters lipid metabolism via the Pparα pathway.

## 1. Introduction

Selenium is an essential trace element that is incorporated into selenoproteins as selenocysteine to mediate its functions. There are 25 and 24 selenoprotein genes in the human and mouse genome, respectively [1,2]. Selenoproteins are primarily involved in redox homeostasis, regulation of signaling cascades, and antioxidant defense [1,2]. However, the precise function of numerous selenoproteins remains unknown [3,4]. Selenium-binding protein 1 (Selenbp1) is a highly conserved and unconventional selenoprotein with distinct (perselenide) or undetermined selenium chemistry [5]. It was first discovered in the mouse liver [6]. The human *SELENBP1* gene is located at 1q21.3, which is homologous to the mouse *Selenbp1* (*SBP56*) gene, which encodes a 56 kDa protein and stably binds with selenium [6,7]. Human SELENBP1 mRNA is ubiquitously expressed, with the highest expression detected in the adult kidney, duodenum, liver, lung, and brain [8]. A recent study demonstrated that mutation of human SELENBP1 can result in halitosis [9]. The transfection of human SELENBP1 into the cell serves as a methanethiol oxidase to form hydrogen sulfide from methanethiol [9]. However, the function of purified SELENBP1 has yet to be confirmed experimentally. Reduced levels of SELENBP1 have been associated with carcinogenesis and poor outcomes in patients [10,11,12]. In contrast, patients with schizophrenia have shown elevated brain SELENBP1 levels [13]. In mice, Selenbp1 is a cytosolic protein that is highly expressed in the liver, kidneys, and gonads [6]. The molecular basis of Selenbp1 needs to be comprehensively elucidated. Several reports have suggested that Selenbp1 is involved in intra-Golgi protein transport [14] and plays important roles in ubiquitination/deubiquitination pathways [15], cell expansion [16], and modulation of oxidative stress [17]. In mice, Selenbp2 is highly homologous to Selenbp1 [18]. In cells, acetaminophen reactive metabolites bind to Selenbp1 along with Selenbp2 [19]. Previous studies have revealed that exposure to 2,3,7,8-tetrachlorodibenzo-*p*-dioxin (TCDD, dioxin) or dioxin-like compounds induce various toxicities in living organisms, including immunosuppression, liver damage, and carcinogenic promotion [20]. Dioxin migrates into the nucleus by binding to the aryl hydrocarbon receptor (AhR), which is localized in the cytosol, forms a heterodimer with the nuclear translocator (Arnt) [21], and binds to a xenobiotic responsive element (XRE) [22]. We previously demonstrated that dioxin-like coplanar polychlorinated biphenyl, 3,3′,4,4′,5-pentachlorobiphenyl (PCB126), and 3-methylcholanthrene significantly induce the expression of Selenbp1 protein in the rat liver [23]. Selenbp1 may be involved in TCDD-induced toxicity, but only a few differences were observed between Selenbp1 knockout (KO) and wild-type (WT) mice in apparent phenotypes, possibly due to the compensatory effect of Selenbp2 on the functions of Selenbp1 [24]. In contrast, our preliminary study suggested that 20 h of fasting did not affect Selenbp1 mRNA expression, but significantly reduced Selenbp2 mRNA expression in the kidney. As the kidney level of Selenbp2 is low [18], we aimed to clarify the role of dioxin-inducible Selenbp1 in the kidney by eliminating other factors altered by dioxin. To address this issue, we performed metabolomic and DNA microarray analyses of the kidneys to examine the effect of Selenbp1 ablation. Based on the observed results, it can be suggested that ablation of Selenbp1 alters the lipid metabolism via downregulation of peroxisome proliferator-activated receptor-α (Pparα (Ppara)).

## 2. Results

### 2.1. Expression Levels of Selenbp1 and Selenbp2 in the Kidney under Fasting and Non-Fasting Conditions

**Figure 1:**
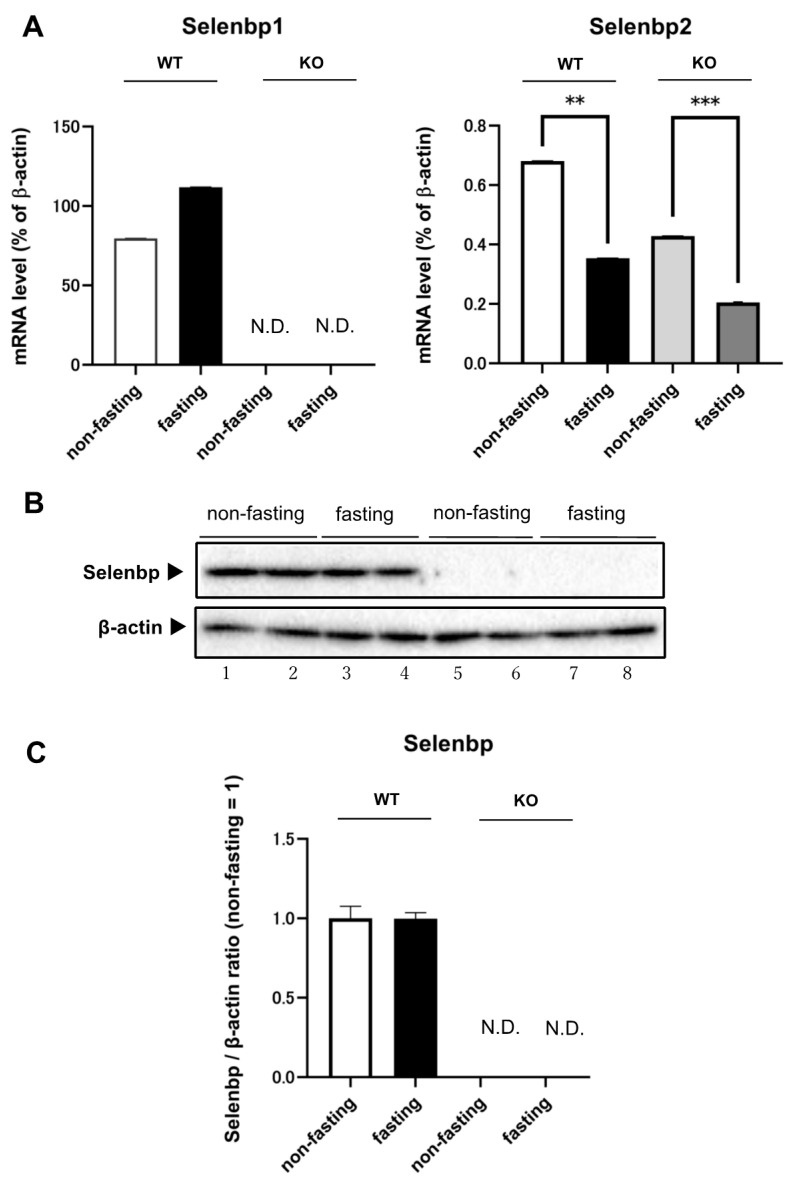
Effect of fasting on the mRNA expression of Selenbp1 and Selenbp2 (**A**) and protein expression levels of Selenbp (**B**,**C**) in the kidney of wild-type and Selenbp1-KO mice. A. The kidneys of 8-week-old male mice were removed. The relative mRNA level was normalized to that of β-actin. Values represent the mean ± standard error of the mean (SEM). Non-fasting (*n* = 6); fasting (*n* = 6). Significant differences from the non-fasting group: ** *p* < 0.01; *** *p* < 0.001. B. The kidneys of 8-week-old male wild-type and Selenbp1-KO mice were removed. Individual kidney S9-fractions (20 μg protein) prepared from wild-type and Selenbp1-KO mice were subjected to SDS-PAGE (10% separation gel). Selenbp was detected using a mouse anti-human Selenbp1 antibody. β-actin was detected using a mouse anti-β-actin antibody. Lanes 1–2, WT non-fasting; 3–4, WT fasting; 5–6, Selenbp1-KO non-fasting; 7–8, Selenbp1-KO fasting. Representative data are presented. Two of the five randomly selected samples were analyzed. C. Values represent the mean ± SEM. Non-fasting (*n* = 5); fasting (*n* = 5). N.D., not detectable; WT, wild-type; KO, knockout; Selenbp1/2, selenium-binding protein 1/2.

In WT and Selenbp1-KO mice, the mRNA expression levels of Selenbp2 considerably differed under fasting conditions when compared with non-fasting conditions. After 20 h of fasting, the mRNA expression level of Selenbp2 was significantly decreased in both WT and Selenbp1-KO mice (Figure 1A). In contrast, the mRNA expression levels of Selenbp1 in the kidney were comparable under fasting and non-fasting conditions. Immunoblotting analysis further showed that Selenbp levels in the kidney were unchanged under fasting and non-fasting conditions in WT mice. However, the protein expression level of Selenbp in the kidneys of Selenbp1-KO mice was undetectable under both fasting and non-fasting conditions (Figure 1B,C). Therefore, the protein band observed in the kidney and cross-reacted with the anti-Selenbp1 antibody is Selenbp1.

### 2.2. Metabolomic Changes in the Kidney of Selenbp1-KO Mice

**Figure 2:**
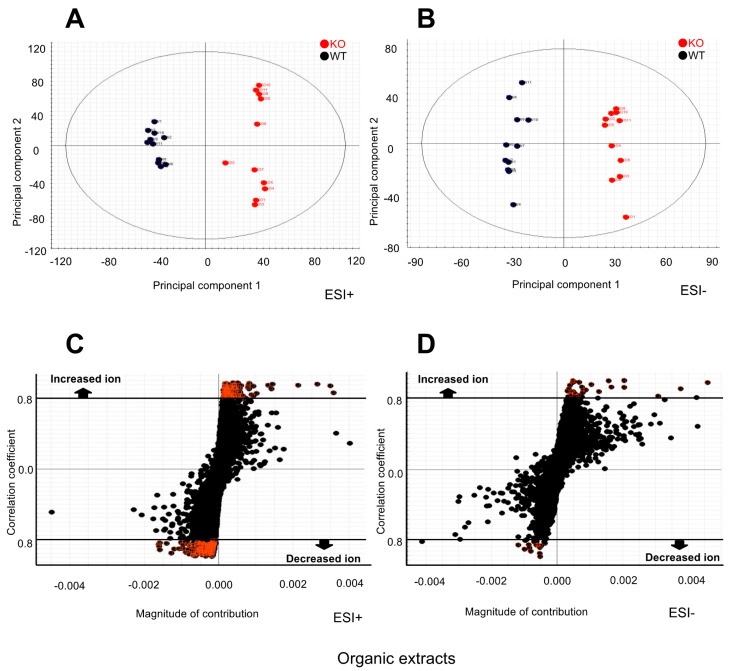
Changes in the renal metabolomic profile of Selenbp1-KO mice compared with the control group using UPLC-TOF/MS. Principal component analysis (PCA) of the effect of Selenbp1 deletion on the kidney metabolome: data from positive (**A**) and negative (**B**) ion mode analysis. Selenbp1 deletion affects the profile of the kidney metabolome. Each dot represents a different animal (*n* = 11 mice per group). Wild-type and Selenbp1-KO mice are shown in black and red, respectively. *S*-plot based on the PCA regarding the effect of Selenbp1 deletion on the kidney metabolome: data from positive (**C**) and negative (**D**) ion mode analysis. Fragment ions in UPLC-TOF-MS analysis are altered by Selenbp1 deletion in the kidneys of male mice (8 weeks old). Each dot represents a single ion with a specific mass (m/z). The criteria for selecting ions significantly altered by Selenbp1 deletion were set either at more than +0.8 or less than −0.8 of the correlation coefficient (red dots). Conditions were under 20 h of fasting. Selenbp1, selenium-binding protein 1; UPLC-TOF/MS, ultra-performance liquid chromatography-time-of-flight mass spectrometry; KO, knockout.

Kidney extracts were subjected to metabolomic analysis using ultra-performance liquid chromatography-time of flight mass spectrometry (UPLC-TOF/MS) to identify prominent metabolite variations, as well as the metabolites responsible for such variations. Based on the principal component analysis (PCA) of the metabolomics data obtained from UPLC-TOF/MS in positive ion and negative mode (Figure 2A,B), the clusters of the plots for the two experimental animal groups were distinctly separated from each other. This observation suggested that the kidney metabolome markedly varied between Selenbp1-KO and WT mice. Further analysis using *S*-plots indicated an increased or decreased number of mass ions fragmented from renal components (Figure 2C,D). In the *S*-plots, compounds with a correlation coefficient smaller than −0.8 or greater than +0.8 are highlighted. The *S*-plot was used to select potential biomarkers and visualize metabolomic changes. The more distant the red dots were, the more likely the biomarker candidates; this could be attributed to their high contributions and correlations. Database information regarding the mass and retention times of these ions suggested that several cellular components were altered, including fatty acid biosynthesis, prostaglandin and leukotriene metabolism, steroid metabolism, fatty acid ester biosynthesis, and ubiquinone biosynthesis. Table 1 shows representative metabolites whose levels were significantly altered, as observed in the metabolomic analyses. Their retention times were determined by monitoring their molecular ions (m/z, [M+H]^+^). Among them, the leukotrienes are metabolites of arachidonic acid derivatives. The immediate product is LTA4 (leukotriene A4), which is converted into either LTB4 (leukotriene B4) by LTA4 hydrolase or LTC4 (leukotriene C4) by LTC4 synthase. LTB4 is a lipid mediator that plays a critical role in acute inflammation [25]. Notably, the present metabolomics showed a reduced level of 20-carboxy-leukotriene B4 (20-carboxy-LTB4) and 20-dihydroxy-leukotriene B4 (20-dihydroxy-LTB4) in the Selenbp1-KO mouse kidney. 20-Carboxy-LTB4 is a member of the class of leukotriene B4, in which the terminal methyl group undergoes formal ω-oxidation to the corresponding carboxylic acid. Additionally, the metabolomic results revealed increased levels of renal prostaglandin J2 (PGJ2) and 11-epi-prostaglandin F2α (11-epi-PGF2α). These prostaglandins are derived from arachidonic acid and are transformed by prostaglandin synthetase into several structurally related carbocyclic molecules [26]. PGJ2 is considered highly neurotoxic when compared with PGA1, D2, and E2 [27]. 11-Epi-PGF2α, derived from PGF2, is responsible for essential biological processes such as inflammation, pain, and sleep. PGF2α is synthesized in several distinct steps. Furthermore, it increases docosapentaenoic acid and 3-hydroxycapric acid. Docosapentaenoic acid (DPA) is an intermediary between eicosapentaenoic acid (EPA, 20:5 ω-3) and docosahexaenoic acid (DHA, 22:6 ω-3). DPA is reportedly associated with thyroid cancer [28], as well as cardiovascular diseases [29]. These lines of evidence suggest that a marked change in the metabolome occurs in the kidney between Selenbp1-KO and WT mice; however, we were unable to perform an in-depth investigation regarding the underlying functional implications, which could improve our understanding of the contributions made by these metabolite alterations on the physiological functions of Selenbp1.

### 2.3. Renal mRNAs in Mice Were Altered by Selenbp1 Deletion via the Pparα Pathway

**Figure 3:**
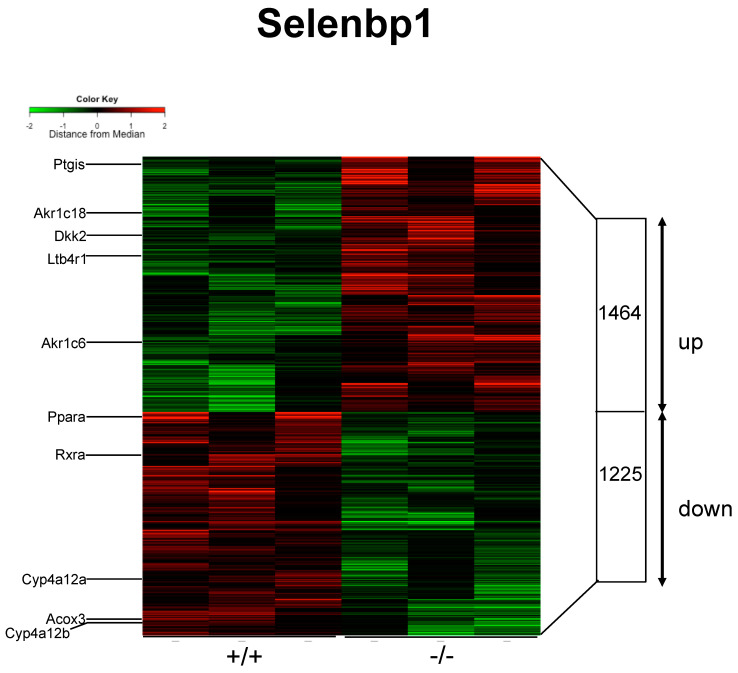
Heatmap of renal mRNAs significantly altered in male adult mice (8 weeks old) by Selenbp1 deletion. Genes whose expression was significantly increased or decreased by Selenbp1 deletion are shown in red and green, respectively. For the magnitude of the alteration, see the color gradation shown in the figure. Each lane is the mRNA prepared from male mice at 8 weeks. Eighteen genes, indicated by arrows and numbers, are shown in Table 2. Conditions under 20 h of fasting. Selenbp1, Selenium-binding protein 1.

**Figure 4:**
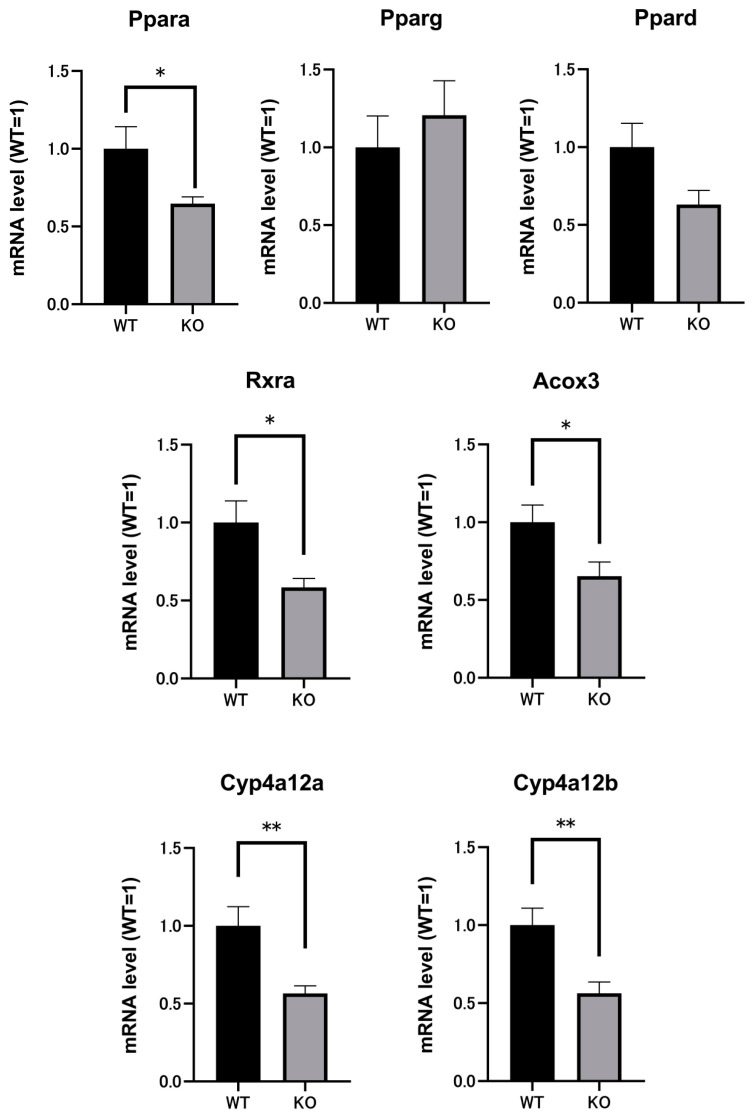
Effect of Selenbp1 ablation on the renal expression of lipid metabolism-related enzymes. The kidneys were collected from 8-week-old male mice fasted for 20 h. The relative mRNA levels of the indicated enzymes were analyzed by real-time RT-PCR and normalized to β-actin mRNA. Each bar represents the mean ± standard error of the mean (SEM) of six mice. β-Actin was used as an internal control. * *p* < 0.05, ** *p* < 0.01. Selenbp1, selenium-binding protein 1; RT-PCR, reverse transcription-polymerase chain reaction.

We comprehensively investigated the genes associated with Selenbp1 deficiency that contributed to its role in physiological functions related to different levels of arachidonic acid derivatives. Accordingly, DNA microarray analysis focusing on the kidney was performed. We expected to find alterations in the genes related to arachidonic acid metabolism and any key significance to elucidate the physiological role of Selenbp1. Microarray-based gene expression profiling was used to identify gene expression changes in response to Selenbp1 gene knockout. In total, 2689 differentially expressed genes were identified; among these, 1464 were increased and 1225 were decreased (Figure 3). These included several representative genes related to lipid and glucose metabolism, such as *Pparα*, retinoid-X-receptor-α (*Rxrα* [*Rxra*]), cytochrome P450 4A12 (*Cyp4a12a*), *Cyp4a12b*, and acyl-CoA oxidase 3 (*Acox3*), which revealed reduced expression in Selenbp1-KO mice (Figure 4). Conversely, ablation of the *Selenbp1* gene increased the expression levels of several other genes, including *Alox5* (arachidonate 5-lipoxygenase) and *Fabp6* (fatty acid-binding protein 6). In addition, the expression of several other genes, such as *Dkk2* (Dickkopf-related protein 2), which is known to be involved in the Wnt signaling pathway, was increased. Next, we attempted to clarify the mechanism via which Selenbp1-KO mice accumulated lipids and arachidonic acid derivatives in the kidney. Hence, we examined the renal expression of mRNAs encoding enzymes that synthesize and metabolize 20-carboxy-LTB4. Cyp4A12a and Cyp4A12b are fatty acid ω- and ω-1 hydroxylases involved in the oxidation of the arachidonic acid derivative leukotriene B4 to 20-carboxy-LTB4 [30]. These ω-hydroxylation pathways have emerged as critical determinants of numerous disease processes, including inflammation and cancer progression [31]. Cyp4a12a and Cyp4a12b are regulated by Pparα, which is a transcription factor belonging to the PPAR subfamily (Pparα, Pparβ/Pparδ (Ppard), Pparγ (Pparg)) and forms a heterodimer with Rxrα [32]. The decreased expression of both Pparα and Rxrα was consistent with the suppressed mRNA levels of Cyp4a12a and Cyp4a12b observed in Selenbp1-KO mice. *Acox3* is a target gene activated by Pparα and is involved in the desaturation of 2-methyl-branched fatty acids in peroxisomes related to lipid metabolism, energy homeostasis, and cell differentiation [33]. Compared with WT mice, the expression level of Acox3 was decreased in Selenbp1-KO mice. Collectively, these results suggested a marked change in the lipid metabolome via the Pparα pathway, which is related to Selenbp1-knockout in the kidney.

### 2.4. SOD mRNA and SOD Activity in the Kidneys of Both Selenbp1-KO and WT Mice

**Figure 5:**
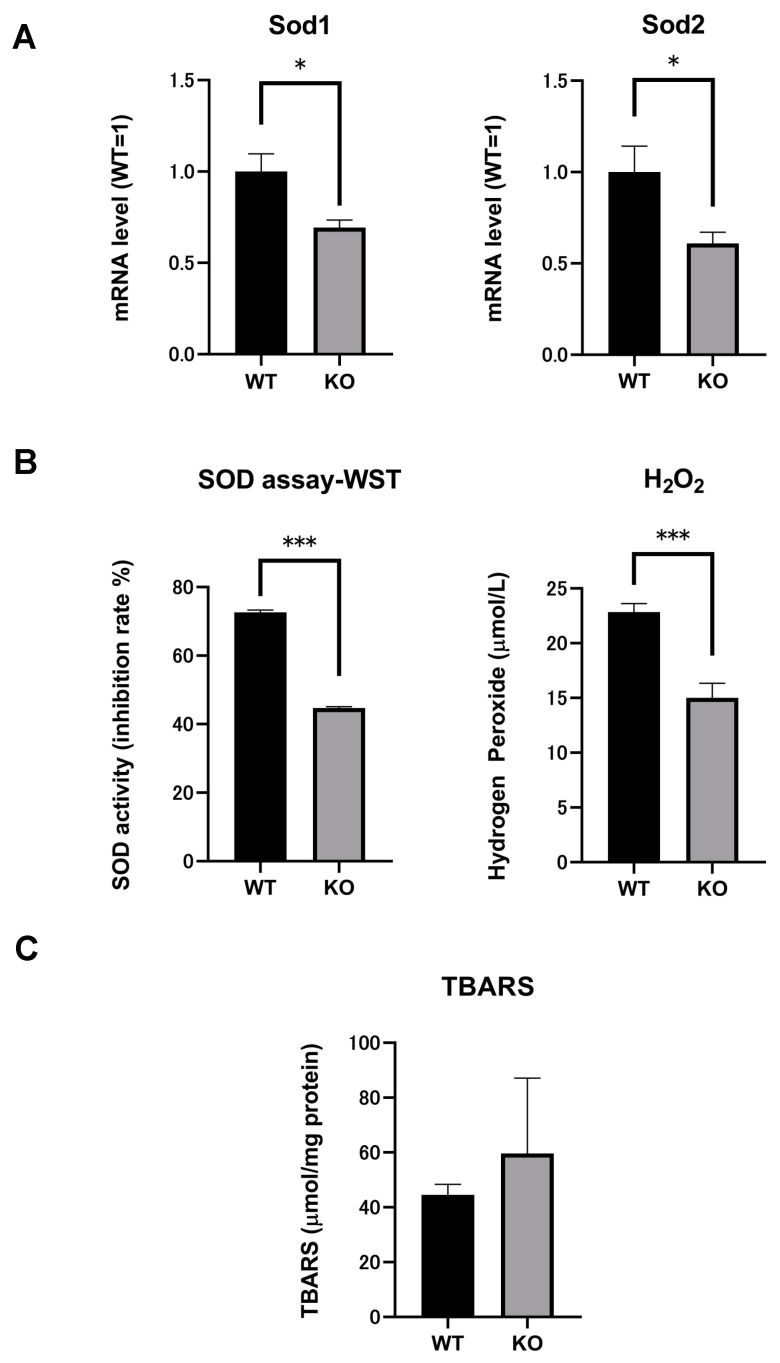
Effects of Selenbp1 on the renal redox reaction. (**A**). Effect of Selenbp1 ablation on the renal expression of oxidative stress-related enzymes. (**B**). SOD activity and hydrogen peroxide concentrations in mice kidneys. SOD activity was determined by a WST1 assay. Hydrogen peroxide concentration was determined by a quantitative peroxide assay. The kidneys were isolated from 8-week-old male mice who were fasted for 20 h. Each bar represents the mean ± standard error of the mean (SEM) of five mice. * *p* < 0.05; *** *p* < 0.001. (**C**). TBARS used as the index of renal lipid peroxidation in Selenbp1-deficient mice. Each bar represents the mean ± SEM of 10 mice. Selenbp1, selenium binding protein 1; SOD, superoxide dismutase; TBARS, thiobarbituric acid reactive substance.

There are three major families of SODs, depending on the metal cofactor. They are classified as copper/zinc SOD (Cu/Zn SOD; SOD1) and manganese SOD (Mn SOD; SOD2). Additionally, an extracellular (EC) form of SOD protein has been identified. EC-SOD (SOD3) mainly occurs in prokaryotes, such as the molting fluid of insects [34]. The mRNA expression levels of Sod1 and Sod2 were significantly lower in the kidneys of 8-week-old Selenbp1-KO mice than in WT mice (*p* < 0.05, Figure 5). Based on the results of the SOD assays, the total SOD activity was significantly lower in the kidneys of Selenbp1-KO mice than in WT mice (*p* < 0.001, Figure 5).

### 2.5. Significant Reductions in H_2_O_2_ Levels Indicated Changes in Oxidation Stress in Selenbp1-Deficient Mice

Oxidative stress leads to altered PPAR expression, occurring via various mechanisms [35]. SODs are major cytoplasmic antioxidant enzymes that metabolize superoxide radicals into molecular oxygen and hydrogen peroxide, affording protection against oxygen toxicity [36]. As Sod1 and Sod2 are Pparα-regulated genes [37], their mRNAs were suppressed in Selenbp1-KO mice (Figure 5A). The level of oxidative stress can be determined by measuring the amount of free radicals, ROS, and antioxidants. As the expression of Sod1 and Sod2 was altered, a quantitative peroxide assay was performed using the kidneys of Selenbp1-KO and WT mice (Figure 5B). Figure 5B shows the quantitative evaluation of hydrogen peroxide in the kidney homogenates of Selenbp1-KO and WT mice. Hydrogen peroxide levels were lower in Selenbp1-KO mice than in WT mice. Based on the reduced SOD activity and Sod1/2 expression, superoxide anions may accumulate in the kidneys of Selenbp1-KO mice.

### 2.6. Comparison of Renal Lipid Peroxidation between Selenbp1-KO and WT Mice

The thiobarbituric acid reactive substance (TBARS), used as an index of lipid peroxidation, mainly measures MDA produced by lipid peroxidation [38]. Figure 5C compares TBARS in the kidneys of Selenbp1-KO and WT mice. Selenbp1 deletion tended to increase TBARS, with no significance. Therefore, the change in oxidative stress caused by Selenbp1 deletion in the kidney is not markedly pathogenic.

### 2.7. Selenium Compounds Were Unaltered in the Kidney and Serum of Selenbp1-KO Mice

The selenium contents in the kidney and serum of Selenbp1-KO and WT mice were determined. The sample tube of the instrument was inserted into the test solution and measured, and the average of three readings was obtained. Next, the corresponding mass concentration from the standard curve was calculated, the corresponding mass concentration of the blank solution was subtracted, and the amount of each element was calculated.

**Figure 6:**
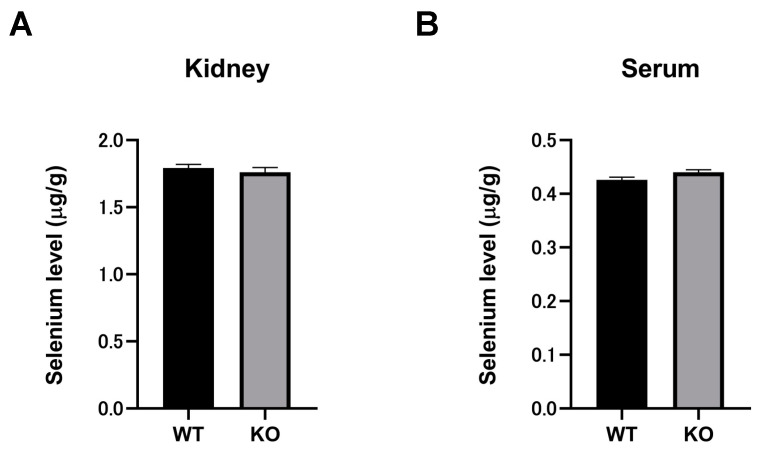
Serum and kidney levels of selenium (µg/g) in wild-type and Selenbp1-KO mice by Agilent Technologies ICP-MS 7700× detection. Each bar represents the mean ± standard error of the mean (SEM) of three mice performed in triplicate: (**A**) kidney; (**B**) serum. Details are described in the Materials and Methods section. Selenbp1, selenium binding protein 1; ICP-MS, inductively coupled plasma mass spectrometry.

The relative standard deviation (RSD) ranged between 0.1% and 2.8%. No significant differences were observed in the kidney and serum selenium content between Selenbp1-KO and WT mice (Figure 6).

## 3. Discussion

Although dioxin induces Selenbp1 in the liver, defining its role has remained challenging because the highly homologous Selenbp2 is also abundantly expressed. In the present study, we evaluated the role of Selenbp1 in the kidney, as Selenbp2 is barely expressed in the kidney. Selenbp1 has been suggested as a novel urinary biomarker for detecting heavy metal-induced nephrotoxicity [39]. It is also worth investigating the potential role of Selenbp1 in detecting early renal damage. Herein, we first analyzed the metabolome of the kidney to clarify the physiological role of dioxin-inducible Selenbp1. In the kidney, the expression of Selenbp2 is reportedly low [18] and fasting significantly reduced Selenbp2 mRNA levels in the absence of dioxin administration (Figure 1A). Furthermore, in the present study, expression of Selenbp2 protein was not detected in the kidneys of Selenbp1-KO mice after 20 h of fasting (Figure 1B). These results prompted us to examine 8-week-old male Selenbp1-KO and C57BL/6J control mice, which were fasted for 20 h before comparison. In the present study, a 20 h fasting period and evaluation of mouse kidneys allowed us to observe in vivo changes induced by Selenbp1 deletion.

As dyslipidemia is a known dioxin-induced toxicity [40], a possible association with Selenbp1 can be postulated. Selenbp1 is significantly induced by dioxins and is related to lipid metabolism. As shown in Table 1, PGJ2 and 11-epi-PGF2α, known as arachidonic acid metabolites, were increased, while the levels of leukotriene metabolites of arachidonic acid, including 20-carboxy-LTB4 and 20-dihydroxy-LTB4, were decreased (Table 1). It is well established that at least three metabolic pathways are involved in the metabolism of arachidonic acid: cyclooxygenase (COX), lipoxygenase (LOX), and cytochrome P450 (CYP) [41,42]. Of these, arachidonic acid is converted into PGH2 by COX-1 or COX-2. PGH2 is then converted to prostaglandin products (PGE2, PGF2, PGD2, PGI2) by specific prostaglandin synthases. PGD2 is highly unstable, resulting in the formation of PGJ2 [43]. In humans, 11-epi-PGF2 is produced by reducing PGD2, which is mediated by aldo-keto reductase family 1 member C3 (AKR1C3), an NADPH-dependent enzyme [44,45]. Previous reports have revealed that aldo-keto reductases influence PGF2 levels and adipocyte differentiation in male mice and humans [46]. In light of these findings, changes in the expression levels of the AKR1C family may induce an elevation in PGF2. Data from real-time RT-PCR and metabolomics substantiate this possibility (Table 1 and Table 2). Additionally, it was revealed that fatty acid metabolism could affect the production of inflammatory substances. PGJ2 is reportedly involved in the pathological changes observed in Alzheimer’s disease and Parkinson’s disease, as well as mediating oxidative stress in neuronal apoptosis and the accumulation/aggregation of ubiquitinated proteins [27,47]. Leukotrienes are potent pro-inflammatory mediators that are synthesized from arachidonic acid. LTB4 is synthesized following the release of arachidonic acid by phospholipase A2 and the enzyme 5-lipoxygenase [48]. In the 5-lipoxygenase pathway, LTA4 is the intermediatory metabolite, transformed to LTB4 by LTA4 hydrolase, while LTC4 synthase catalyzes LTC4 formation [49]. In human neutrophils, the metabolism of LTB4 by a specific cytochrome P450-dependent pathway (CYP4F3) produces 20-hydroxy-LTB4 [50]. The initial ω-oxidized metabolite 20-hydroxy-LTB4 subsequently yields inactive 20-carboxy-LTB4 via both a P-450-dependent pathway and an alcohol/aldehyde dehydrogenase-dependent pathway in certain cells [51,52]. Therefore, the decreased metabolites of 20-hydroxy-LTB4 and 20-carboxy-LTB4 in Selenbp1-KO mice may reverse the increase in LTB4 observed in the kidney. The P450 pathway is the major metabolic pathway for arachidonic acid metabolism in the kidneys [53]. The CYP4A subfamily members Cyp4a12a and Cyp4a12b are expressed in the mouse kidney and catalyze the ω-hydroxylation of arachidonic acid [54]. In the present study, we revealed that the renal mRNA expression levels of Cyp4a12a and Cyp4a12b were decreased in Selenbp1-KO mice when compared with the control animals (Figure 4). A recent report indicated that transgenic CYP4A12 mice developed salt-resistant hypertension, which was attributed to the increase in 20-hydroxyeicosatetraenoic acid (20-HETE) in the renal and blood vessels, including oxidative stress, endothelial dysfunction, and increased vascular reactivity [55]. This suggests that unregulated CYP4A12 may cause kidney damage, including inflammation, via arachidonic acid metabolism [56]. Reportedly, CYP4A induction is regulated through a member of the nuclear receptor superfamily named PPAR, which are ligand-activated transcription factors [57]. There are three isotypes of Ppar: Pparα, Pparβ(δ), and Pparγ, each displaying a different expression pattern [58]. Recent studies have discussed the role of Ppar in diabetes, cancer, hypertension, inflammatory disease, and kidney disease [59]. Cyp4a12a and 12b are positively regulated by Pparα [60]. Moreover, as the expression level of Pparα decreased following the knockout of Selenbp1 (Figure 4), it can be suggested that the decreased expression of Pparα mediated the reduced expression of Cyp4a12a and Cyp4a12b. The mRNA expression of Rxrα was also reduced in the Selenbp1-KO mice (Figure 4). Rxrα forms heterodimers with several nuclear receptors, including liver X receptor (Lxr), pregnane X receptor (Pxr), retinoic acid receptor (Rar), constitutive androstane receptor (Car), and Ppar and is involved in transcriptional regulation [61]. In the present study, we focused on the effect on lipid metabolism, investigating the effect of Selenbp1 deletion on the relevant transcription factors. Acox3, which is suppressed by Selenbp1 deletion (Figure 4), is also known as peroxisomal acyl-coenzyme A oxidase 3 and is involved in the desaturation of 2-methyl branched fatty acids in peroxisomes [62]. Branched-chain fatty acids are thought to be derived from branched-chain amino acids such as valine, leucine, and isoleucine [63]. As the expression of Acox3 was significantly reduced (Figure 4), this finding is consistent with the reduced expression of Pparα and Rxrα, as several of the enzymes involved in peroxisomal oxidation are under the control of Pparα. Conversely, metabolomic analyses suggested an increase in prostaglandins, with almost no effect of Selenbp1-KO on these synthetic enzymes observed (Supplementary Figure S1). Therefore, it was suggested that prostaglandins were increased by suppressing the Cyp4a12a and Cyp4a12b systems. Therefore, future in-depth investigations in terms of inflammation are necessary. In addition to the DNA microarray data, metabolomics also indicated that Selenbp1 is at least involved in lipid metabolism in the kidney; however, its deficiency does not have a lethal effect on the entire body. Furthermore, it can be suggested that Selenbp1 deficiency may cause metabolic switching, namely, suppressing Cyp4a-mediated fatty acid oxidation and increasing inflammatory biotransformations. Based on these findings, the linkage between Selenbp1 and lipid metabolism is presumably attributed to its effect on Pparα and Rxrα expression.

Conversely, in human cancer cells, i.e., HeLa cells, in vitro knockdown of Selenbp1 by shRNA resulted in reduced sensitivity toward exogenous excess hydrogen peroxide [64]. A previous report has suggested the role of Selenbp1 in oxidative stress, but the underlying mechanism remains elusive. Furthermore, the conditions used were drastic and considerably differed from physiological conditions. In the present study, we revealed that the knockout of Selenbp1 reduced the expression levels of the antioxidant enzymes Sod1 and Sod2. Reportedly, Pparα induces Sod1 and Sod2 [65], which is consistent with the suppressed Pparα expression mediated by Selenbp1 knockout in the present study. Furthermore, the decreased hydrogen peroxide and SOD activity in the kidneys of Selenbp1-KO mice possibly increased the accumulation of superoxide anions and induced changes in lipid metabolism. Herein, the TBARS assay did not show a significant difference between Selenbp1-KO and WT mice, but potential oxidative stress may attack nucleic acids, cause protein damage, thus impairing cell function [66]. Although limited data are currently available regarding the kidney, it is reasonable to suppose that constitutively expressed Selenbp1 is an important modulator of oxidative stress and lipid metabolism by modulating Pparα and Rxrα expression. Given its crucial role, the induction of Selenbp1 by dioxin may counteract its acute toxicity on lipid metabolism, such as the accumulation of LTB4 [67]. Another possibility is that the induction of Selebp1 by dioxin disturbs its physiological function in lipid metabolism. Although the mechanism via which Selenbp1 deficiency reduces Pparα and Rxrα expression remains unclear, the fluctuating status of oxidative stress needs to be assessed in future studies. Reportedly, the cellular redox state and oxidative stress may serve as essential transcriptional regulators for Pparα [68]. Thus, the possibility that Selenbp1 regulates oxidative stress to alter Pparα expression needs to be explored in future research.

In conclusion, our findings can be summarized as follows. (1) Metabolomic analysis suggests that constitutively expressed Selenbp1 is associated with lipid metabolism. (2) Decreased expression of oxidative enzymes such as Cyp4a12a, Cyp4a12b, and Acox3 in the kidney was confirmed by Selenbp1 knockout under fasting conditions. Reduced expression of Pparα and Rxrα was observed following ablation of Selenbp1, which is involved in the expression of these genes. (3) Alox and prostaglandin-endoperoxide synthase were not affected, but prostaglandins and leukotrienes were increased. (4) The reduction of the active oxygen-scavenging enzymes Sod1 and Sod2 resulted in decreased hydrogen peroxide levels, thereby increasing superoxide anions in the kidneys of Selenbp1-KO mice. These results unquestionably demonstrate that the role of Selenbp1 is associated with lipid metabolism through the Pparα pathway and oxidative stress. Accordingly, it can be suggested that Selenbp1 promotes fatty acid oxidation and plays a suppressive role in inflammation. Further studies are warranted to elucidate the detailed role of Selenbp1.

## 4. Materials and Methods

### 4.1. Animals and Treatments

All animal experiments were approved by the Institutional Animal Care and Experimental Committee of Kyushu University, Japan. Seven-week-old C57BL/6J male mice were obtained from CLEA Japan (Tokyo, Japan). The generation of Selenbp1-KO mice has been previously described [24]. Mice were fed a standard chow (CE-2; CLEA Japan), provided with sterilized water ad libitum, and maintained in an environmentally controlled room at 22 ± 5 °C and 50 ± 15% relative humidity under a 12 h/12 h light/dark cycle (light period, 7:00 a.m.–7:00 p.m.). Both C57BL/6J and Selenbp1-KO mice were acclimatized until 8 weeks of age and were then fasted for 20 h. The animals were anesthetized by CO_2_ inhalation and sacrificed. The whole left kidney was rapidly removed, snap-frozen in liquid nitrogen, and stored at −80 °C.

### 4.2. Immunoblotting

The collected tissues were homogenized using a glass-Teflon homogenizer in 0.25 M sucrose, which corresponded to 3 times the weight of the kidney. The obtained homogenate was centrifuged at 9000× *g* at 4 °C for 20 min, and the supernatant (S9) was obtained. The protein concentration was determined according to Lowry et al. [69]. Protein samples (20 μg) underwent electrophoresis on a 7.5% sodium dodecyl sulfate (SDS)-polyacrylamide gel, followed by transfer to polyvinylidene difluoride (PVDF) membranes (Merck KGaA-Millipore, Burlington, MA, USA). The target protein was treated with the primary antibody overnight and incubated with a secondary antibody conjugated to horseradish peroxidase (HRP) for 60 min at room temperature. The membrane was then washed with TBS-Triton and immersed in a Clarity Western ECL substrate for 5 min at room temperature. Relative levels of target proteins were determined using a ChemDoc system operated by Image Lab software (Bio-Rad, Hercules, CA, USA) in auto-exposure mode. β-Actin was used as a loading control for Selenbp1 and Selenbp2.

### 4.3. Metabolomics of Kidneys

In brief, mouse kidney samples (50 mg) were homogenized in 1.5 mL of cold methanol/water (1:1, *v/v*) and centrifuged at 11,000× *g* for 10 min. The solid precipitates were homogenized in 1.5 mL of cold dichloromethane/methanol (3:1, *v/v*) and centrifuged at 11,000× *g* for 10 min. The supernatant was evaporated to dryness (organic extract) under a nitrogen stream. The operation conditions for UPLC and detailed procedures followed the manual for 2-step solvent extraction of tissue samples [70]. The organic extracts were finally reconstituted in 150 μL methanol/water (1:1, *v/v*), and 10 μL organic extracts of the solution were subjected to metabolomic analysis using UPLC-TOF/MS (LCT-premier XE; Waters, Milford, MA, USA). Chromatographic separation of the organic tissue extract was performed on a BEH C8 column (2.1 × 100 mm, 1.7 μm; Waters) using Waters’ ACQUITY UPLC System (Waters) [71]. First, the data were subjected to principal component analysis (PCA). To compare metabolomic profiles between control and Selenbp1-KO groups, data were analyzed using an orthogonal partial least squares discriminant analysis (OPLS-DA) method [72]. From the *S*-plot obtained, fragment ions with correlation coefficients of more than +0.8 and less than −0.8 were extracted as ions significantly increased and decreased by Selenbp1 deletion, respectively. Second, components altered by control and Selenbp1-KO were estimated by referring to the retention time and mass information of the ions in online databases, including the Human Metabolome Database (http://www.hmdb.ca/ at 14 November 2019) and the Kyoto Encyclopedia of Gene and Genomes (KEGG, http://www.genome.jp/ at 15 November 2019).

### 4.4. DNA Microarray Analysis

The left kidneys were excised from male Selenbp1-KO and WT mice, and each isolated sample was rapidly placed into a tube filled with RNAlater and used for RNA extraction without freezing. Total RNA was extracted using a RNeasy Mini Kit (Qiagen, GmbH, Hilden, Germany). RNA was purified by ethanol precipitation and dissolved in RNase-free water. RNA passing the quality check was subjected to DNA microarray analysis. Total RNA was converted to cyanine-labeled cRNA using a Low-Input QuickAmp Labeling kit (Agilent Technologies, Santa Clara, CA, USA), according to the manufacturer’s instructions. Dye incorporation and cRNA yield were determined using a NanoDrop ND-1000 spectrophotometer. Next, 1500 ng cyanine-labeled cRNA was overlaid onto individual microarrays immobilized with 41,000 mouse gene transcripts (Agilent). The data obtained were converted to figures using Feature Extraction software (Agilent). Genes agreeing with the criterion that their expression was detected in 1 or more samples out of a total of 6 samples, including 3 WT and 3 KO mice, at detection *p*-values less than 0.05, were selected and further analyzed. Next, to identify any significant differences, the data were processed using a package in the Bioconductor, i.e., Linear Models for Microarray Analysis (Limma) [73], and the criterion for a significant difference between the Selenbp1-KO and WT mice was set at Limma *p* < 0.05. The microarray datasets were submitted to the Gene Expression Omnibus (GEO) database (accession number: GSE169517; www.ncbi.nlm.nih.gov/geo/provided, accessed on 24 March 2021, in the public domain by NCBI). *p*-values of ˂0.05 were selected and subjected to functional annotation analysis using the Database for Annotation, Visualization, and Integrated Discovery (DAVID) (DAVID Bioinformatics Resources 6.8; online at http://david.ncifcrf.gov/, 20 February 2018).

### 4.5. Quantitative Reverse Transcription (RT)-Polymerase Chain Reaction (PCR)

mRNA expression was quantified by real-time RT-PCR as previously reported [74]. Primer sequences are shown in the Supplementary Table S1. Briefly, total RNA was extracted from the left kidney using RNeasy kits (Qiagen) and reverse-transcribed to cDNA. The cDNAs were synthesized using the PrimeScript RT reagent kit with gDNA Eraser (Perfect Real Time, TaKaRa-Bio, Shiga, Japan). The obtained RNA (150 ng) was treated with gDNA Eraser to digest contaminating genomic DNA and then reverse-transcribed to synthesize cDNA. cDNA was amplified with Fast SYBR Green Master Mix (Thermo Fisher Scientific, Inc., Waltham, MA, USA), using the StepOnePlus Real-time PCR system (Thermo Fisher Scientific). The PCR conditions were as follows: 95 °C for 20 s, 50 cycles of 95 °C for 3 s, and 60 °C for 30 s. The relative mRNA levels were determined using the 2^−∆CT^ method. The amount of quantified target mRNAs was normalized to β-actin and presented as a ratio to the WT control.

### 4.6. Superoxide Dismutase Assay with the Xanthine Oxidase/WST System

The SOD assay kit-WST was purchased from Dojindo Molecular Technologies (Dojindo, Kumamoto, Japan). Approximately 50 mg of kidney tissue was used from both WT and Selenbp1-KO mice. For sample homogenization, 6 times the tissue weight of sucrose buffer (0.25 M sucrose, 10 mM Tris, 1 mM EDTA; pH 7.4) was added (*n* = 6 for each group). Homogenized tissues were centrifuged at 10,000× *g* at 4 °C for 60 min, and the supernatant was diluted with saline at ×(1/5)3 to prepare the sample solution. Using 96-well plates, samples and blanks were assessed according to the following protocol. The 96-well plate was incubated at 37 °C for 20 min, and the absorbance was measured at 450 nm using a microplate reader. Finally, the SOD activity (inhibition rate, %) was calculated.

### 4.7. Detection of Hydrogen Peroxide

The quantification of H_2_O_2_ was based on a ferrous oxidation/xylenol orange assay using the Pierce Quantitative Peroxide assay according to the manufacturer’s instructions (Thermo Fisher Scientific). Briefly, 50 mg of the mouse kidney tissue was weighed and homogenized in homogenization buffer with 500 nM Tris-HCl (pH 7.0), 1% protein inhibitor cocktail (cOmplete Tablets EASYpack, Roche, Mannheim, Germany), and 1% NP-40 (KO: 6; WT: 6). After centrifugation at 9500× *g* and 4 °C for 15 min, an aliquot of the supernatant was transferred to a tube and protein was measured according to Lowry et al. [69], using bovine serum albumin as the standard. After the sample protein concentration was adjusted to 264 mg/mL, the hydrogen peroxide concentration was determined. Test tubes containing 100 μL of the sample solution and 1 mL of the working reagent solution were maintained at 30 °C for 30 min and then immediately measured at a wavelength of 560 nm using a spectrometer. Absorbance values were calibrated to a standard curve generated using a known concentration of H_2_O_2_.

### 4.8. Assay for Peroxides in Mouse Kidneys Using the Thiobarbituric Acid Reaction

In brief, the left kidney was homogenized in 1.15% KCl using a homogenizer. Homogenates were prepared at a ratio of 1 g of wet tissue to 9 mL of 1.15% KCl. The protocol was performed as previously reported [38]. The level of lipid peroxides was expressed as μmoL of malondialdehyde (MDA). A 150 μM tetramethoxypropane (TMP) aqueous solution was prepared and serially diluted to prepare a dilution series. TMP was substituted with 10% renal homogenate in the reaction, and the absorbance was measured in the same manner to obtain the absorbance curve. The concentration of MDA in each sample was determined using an absorbance curve.

### 4.9. Quantification of Selenium Compounds by Inductively Coupled Plasma Mass Spectrometry (ICP-MS)

The determination of selenium contents in the kidney and serum was performed by IDEA Consultants, Inc. (Osaka, Japan). To detect the total selenium content in mouse kidneys (KO: *n* = 3; WT: *n* = 3) and serum (KO: *n* = 3; WT: *n* = 3), an ICP-MS method was established (ICP-MS 7700x, Agilent Technologies, Santa Clara, CA). The left kidney was weighed using a precision balance, placed in a Teflon container, and wet-decomposed with nitric acid (Nitric acid 1.42 Ultrapur-100; 28163-5B, Kanto Chemical, Tokyo, Japan). Yttrium (PLY2-2M, SPEX CertiPrep, Metuchen, NJ, USA) was added as an internal standard to the volume. Next, 0.1 mL of serum was taken, yttrium was added as an internal standard, and the volume was adjusted with a solution containing a surfactant. The selenium content was determined by ICP-MS. All chemicals used were of analytical grade, and a standard selenium solution (192-13861, Fuji Film Wako, Osaka, Japan) was used exclusively.

### 4.10. Statistical Analysis

The statistical differences between the 2 experimental groups were compared by Student’s *t-*test using GraphPad Prism 8 (GraphPad Software, San Diego, CA, USA). Statistical significance was set at *p* < 0.05.

## Figures and Tables

**Table 1:** Suggested components related to lipid metabolism and altered levels in the kidneys of male Selenbp1-KO mice by metabolomics analysis.

Sample	Metabolic Pathways	Retention Time (min)	m/z	Metabolite	X-Fold	*p*-Value
Selenbp1-KO/Wild-type	Steroid metabolism	1.56 1.42	347.1458 345.1872	19-Noraldosterone 11-Dehydrocorticosterone	3.6 12.4	0.0115 0.0073
	Prostaglandin and leukotriene metabolism	6.89 1.04	335.1496 355.1588	Prostaglandin J2 11-Epi-prostaglandin F2α	3.7 4.6	0.0161 0.0056
	Fatty acid biosynthesis	1.43 1.05	331.2071 303.0772	Docosapentaenoic acid 3-Hydroxycapric acid	1.7 1.8	0.0139 0.0335
	Fatty acid ester biosynthesis	1.71	435.3324	Butyl oleate sulfate	24.0	0.0498
	Ubiquinone biosynthesis	2.01	451.3326	Vitamin K1	5.0	0.0156
Wild-type /Selenbp1 -KO	Sex hormone metabolism	1.33	289.1626	4-Hydroxyestradiol	4.0	0.0269
	Prostaglandin and leukotriene metabolism	1.41 1.63	367.1941 353.181	20-Carboxy-leukotriene B4 20-Dihydroxy-leukotriene B4	16.9 4.4	0.0128 0.0165
	Steroid lipid	1.07	585.3781	Cholic acid glucuronide	4.0	0.0042

**Table 2:** Suggested genes related to lipid metabolism and significantly altered following ablation of Selenbp1 by DNA microarray analysis.

	Name	Gene ID	Comparison Ratio	*p*-Value
1	Rxrα (retinoid X receptor alpha)	20181	0.873	0.0261
2	Acox3 (acyl-coenzyme A oxidase 3)	80911	0.688	0.0017
3	Cyp4a12a (cytochrome P450, family 4)	277753	0.684	0.0042
4	Cyp4a12b (cytochrome P450, family 4)	13118	0.672	0.0029
5	Pparα (peroxisome proliferator-activated receptor alpha)	19013	0.792	0.0297
6	Cpt1a (carnitine palmitoyl transferase 1a)	12894	0.834	0.0194
7	Cyp2e1 (cytochrome P450, family 2, subfamily e, polypeptide 1)	13106	0.883	0.0488
8	Cyp2a4 (cytochrome P450, family 2, subfamily a, polypeptide 4)	13086	0.741	0.0110
9	Cyp2a5 (cytochrome P450, family 2, subfamily a, polypeptide 5)	13087	0.736	0.0078
10	Slc51a (solute carrier family 51, alpha subunit)	106407	0.864	0.0290
11	Fads2 (fatty acid desaturase 2)	56473	0.518	0.0037
12	Dkk2 (dickkopf homolog 2)	56811	1.337	0.0048
13	Bpifa1 (BPI fold containing family A member 1)	18843	1.190	0.0453
14	Fabp6 (fatty acid-binding protein 6)	16204	2.236	0.0420
15	Lpcat2 (lysophosphatidylcholine acyltransferase 2)	270084	1.240	0.0423
16	Alox5 (arachidonate 5-lipoxygenase)	11689	1.949	0.0194
17	Ltb4r1 (leukotriene B4 receptor1)	16995	1.372	0.0287
18	Ptgis (prostaglandin I2 synthase)	19223	1.359	0.0374
19	Ptges3l (prostaglandin E synthase 3-like)	56351	1.267	0.0209
20	Akr1c6 (aldo-keto reductase family 1 member C6)	83702	1.746	0.0442
21	Akr1c14 (aldo-keto-reductase family 1 member C14)	105387	1.144	0.0460
22	Akr1c18 (aldo-keto-reductase family 1 member C18)	105349	1.520	0.0057
23	Akr1c 20 (aldo-keto-reductase family 1 member C20)	116852	1.367	0.0115
24	Slc25a23 (solute carrier family 25, member 23)	66972	1.221	0.0184
25	Slc25a19 (solute carrier family 25, member 19)	67283	1.182	0.0316
26	Slc2a3 (solute carrier family, member 3)	20527	1.723	0.0167
27	Slc11a1(solute carrier family 11, member 1)	18173	1.295	0.0058
28	Pltp (phospholipid transfer protein)	18830	1.534	0.0452
29	Acsbg1 (acyl-CoA synthetase bubblegum family member 1)	94180	1.302	0.0073
30	Angptl6 (angiopoietin-like 6)	70726	1.221	0.0157

## Data Availability

The microarray datasets were submitted to the Gene Expression Omnibus (GEO) database (accession number: GSE169517; www.ncbi.nlm.nih.gov/geo/, 24 March 2021, provided in the public domain by NCBI).

## Supplementary Materials

The following are available online at https://www.mdpi.com/article/10.3390/ijms22105334/s1. Table S1: Primer sequences used for the PCR amplification of mRNAs. Figure S1: Effect of Selenbp1 ablation on renal expression involved in the arachidonic acid (AA) metabolism-related cyclooxygenase (COX) and lipoxygenase (LOX) enzymes.
