# Supplementary material for: Ablation of Selenbp1 Alters Lipid Metabolism via the Pparα Pathway in Mouse Kidney
Source: Int J Mol Sci. 2021 May 19;22(10):5334. doi: 10.3390/ijms22105334 (PMC8159118; doi:10.3390/ijms22105334)
Supplement: Supplementary file 1 [file ijms-22-05334-s001.zip › ijms-1189421-supplementary.pdf]

International Journal of Molecular Science

Ablation of Selenbp1 alters lipid metabolism via the Pparα pathway in mouse kidney

Yingxia Song[1,2], Atsushi Kurose[1,2], Renshi Li[1], Tomoki Takeda[1], Yuko Onomura[1], Takayuki Koga[3], Junpei Mutoh[4], Takumi Ishida[5], Yoshitaka Tanaka[2] and Yuji Ishii[1,2,*]

1. Laboratory of Molecular Life Sciences, Graduate School of Pharmaceutical Sciences, Kyushu University, 3-1-1 Maidashi, Higashi-ku, Fukuoka 812-8582, Japan
2. Division of Pharmaceutical Cell Biology, Graduate School of Pharmaceutical Sciences, Kyushu University, 3-1-1 Maidashi, Higashi-ku, Fukuoka 812-8582
3. Laboratory of Hygienic Chemistry, Daiichi University of Pharmacy, 22-1 Tamagawa-cho, Minami-ku, Fukuoka 815-8511, Japan
4. Faculty of Pharmaceutical Sciences, Sanyo-Onoda City University, Daigakudori 1-1-1, Sanyo-Onoda, Yamaguchi 756-0884, Japan
5. School of Pharmacy, International University of Health and Welfare Fukuoka, Ohkawa, Fukuoka 831-8501, Japan

* To whom correspondence should be addressed to:

Yuji Ishii, Ph.D.

Division of Pharmaceutical Cell Biology, Graduate School of Pharmaceutical Sciences, Kyushu University, 3-1-1 Maidashi, Higashi-ku, Fukuoka, 812-8582, Japan

Phone: +81-92-642-6586, Fax: +81-92-642-6588, E-mail: ishii@phar.kyushu-u.ac.jp

Supplementary Table S1

Primer sequences used for the PCR amplification of mRNAs

Target mRNA (Genebank Accession)	Primer sequence	Product size
Selenbp1 (NM_009150)	Forward: 5’-CTGATACTGCCTGGTCTCA-3’<br>Reverse: 5’-AGTGGCTGGTGTGCAAAC-3’	142 bp
Selenbp2 (NM_019414)	Forward: 5’-CTGATACTGCCTGGTCTCA-3’<br>Reverse: 5’-AGTGGCTGGTGTGCGTAT-3’	142 bp
Ppara (NM_011144)	Forward: 5’-CATCACAGACACCCTCTCTC-3’<br>Reverse: 5’-AAGCCCTTACAGCCTTCAC-3’	174 bp
Pparg (NM_011146)	Forward: 5’-AGACCACTCGCATTCCTTTGAC-3’<br>Reverse: 5’-TTTATCCCCACAGACTCGGCAC-3’	274 bp
Ppard (NM_011145)	Forward: 5’-AACACACGTTCCTTCCAG-3’<br>Reverse: 5’-GATCGCACTTCTCATACTCG-3’	237 bp
Rxra (NM_011305)	Forward: 5’-CTCCTTCACCAAGCACATC-3’<br>Reverse: 5’-GTCTTTGCGTACTGTCCTC-3’	115 bp
Cyp4a12a: (NM_177406)	Forward: 5’-GACTTCTATCACCTGGAATGAC-3’<br>Reverse: 5’-AGCTCTCTGCTCACACTTG-3’	105 bp
Cyp4a12b (NM_172306)	Forward: 5’-TACTCAGCAGTTCCCATCC-3’<br>Reverse: 5’-TCTCCCCAGAATCAGCTTC-3’	187 bp
Acox3 (NM_030721)	Forward: 5’-GGACAGGACTGGGAATATCAC-3’<br>Reverse: 5’-CAGACATGCTGATGATGGAG-3’	125 bp
Sod1 (NM_011434)	Forward: 5’-ATGGGTTCCACGTCCATCAG-3’<br>Reverse: 5’-GTCTCCAACATGCCTCTCTTC-3’	122 bp
Sod2 (NM_013671)	Forward: 5’-ACAACTCAGGTCGCTCTTC-3’<br>Reverse: 5’-ATAGCCTCCAGCAACTCTC-3’	128 bp
β-actin (NM_007393)	Forward: 5’-GATTACTGCTCTGGCTCCTA-3’<br>Reverse: 5’-TCCTGCTTGCTGATCCAC-3’	135 bp

Supplementary Figure S1

lipoxygenase (LOX)

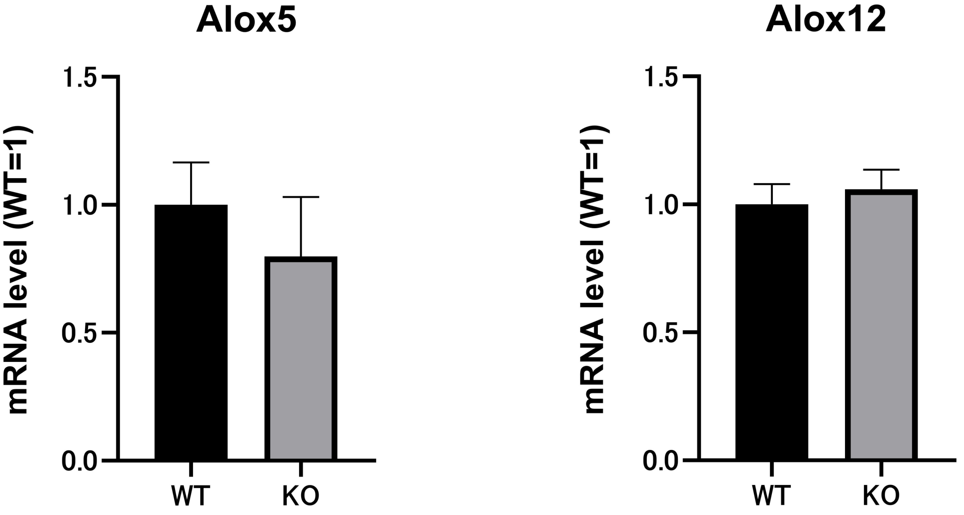

Cyclooxygenase (COX)

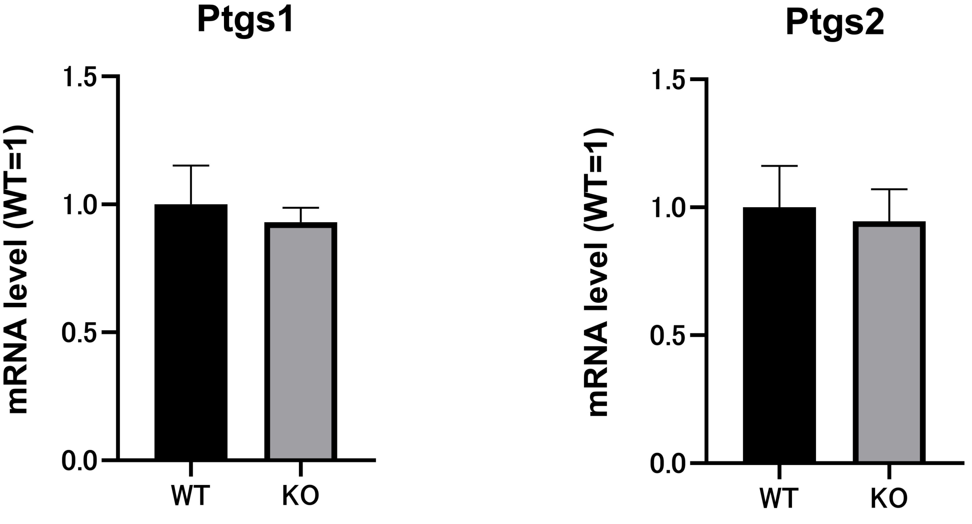

Figure S1. Effect of Selenbp1 ablation on the renal expression involved in the arachidonic acid metabolism related cyclooxygenase (COX) and lipoxygenase (LOX) enzymes.
The kidneys were collected from 8-week old male mice which treated with 20 h fasting. The relative levels of mRNAs indicated were analysis by real-time RT-RCR and normalized by β–actin mRNA. Each bar represents the mean ± S.E.M. of 6 mice. β-actin was used as an internal control. Abbreviations used: Alox, arachidonate lipoxygenase; Ptgs, prostaglandin-endoperoxide synthase.
